# Supplementary material for: Magnetic sensitivity enhancement via polarimetric excitation and detection of an ensemble of NV centers
Source: Sci Rep. 2024 May 23;14:11793. doi: 10.1038/s41598-024-60199-z (PMC11116463; doi:10.1038/s41598-024-60199-z)
Supplement: Supplementary file 1 — Supplementary Information. [file 41598_2024_60199_MOESM1_ESM.pdf]

Magnetic sensitivity enhancement via polarimetric excitation and detection of an ensemble of NV centers: Supplementary material

Simone Magaletti[1], Ludovic Mayer[1], Xuan Phuc Le[1], and Thierry Debuisschert[1,*]

[1]Thales Research and Technology, 91767 Palaiseau Cedex, France.
[*]thierry.debuisschert@thalesgroup.com

1 Laser Excitation and PL collection from {100}, {110} and {111} planes

We report on the results of simulations where the diamond is excited and its PL is collected from a {100} plane (Supplementary figure 1a-d), a {110} plane (Supplementary figure 1e-h) and a {111} plane (Supplementary figure 1i-l), as it usually happens in experiments based on confocal microscopy.

In Supplementary figure 1b we show the excitation probability and equivalently the PL collection efficiency respectively for different angles of laser polarization and polarizer axis orientation. They are maximum when the laser polarization/polarizer axis is perpendicular to the NV center axis. They are minimum, but never zero, when the laser polarization/polarizer axis is oriented along the NV center projections on the excitation/imaging plane. As expected from symmetry reasons, NV centers families A-B and C-D have the same behaviour. Supplementary figure 1c shows the dependence of R_i with the polarizer axis orientation when the laser is polarized along the projection of family A and B on the {100} plane (45°). We observe that the contribution of these two families can be almost suppressed (A and B) while the other two equally contribute to the total PL (R_i=0.45). A similar behaviour is followed also by χ_i (Supplementary figure 1d), confirming the major role of contrast versus total PL on the NV center magnetic sensitivity.

Concerning the excitation and PL detection from a {110} plane (Supplementary figure 1e), we can completely suppress the contribution of one of the two NV center families laying on the {110} plane to the total PL, that is family C and D (Supplementary figure 1f), simply aligning the laser polarization or the polarizer axis to the NV center axis. Supplementary figure 1g shows the dependence of R_i with the orientation of the polarizer axis when the laser is polarized along family D, which is therefore not excited. As discussed in the main text, we can observe that the maximum value of R_i for the three other families is not achieved for the same polarizer orientations that maximize their PL collection efficiency (Supplementary figure 1f). This happens because the maximization of R_i, and thus of the ODMR contrast of family i, not only depends on the PL collection efficiency of family i but also on the PL emitted by the other families. The dependence of χ_i on the polarizer angle, for the same laser polarization as before, is plotted in Supplementary figure 1h and it follows the behaviour of R_i. It is interesting to remark that the polarizer angle that maximizes χ_i does not coincide with the one that maximizes R_i. This is due to the contribution, though minimal, of the total detected PL (S_0) to sensitivity.

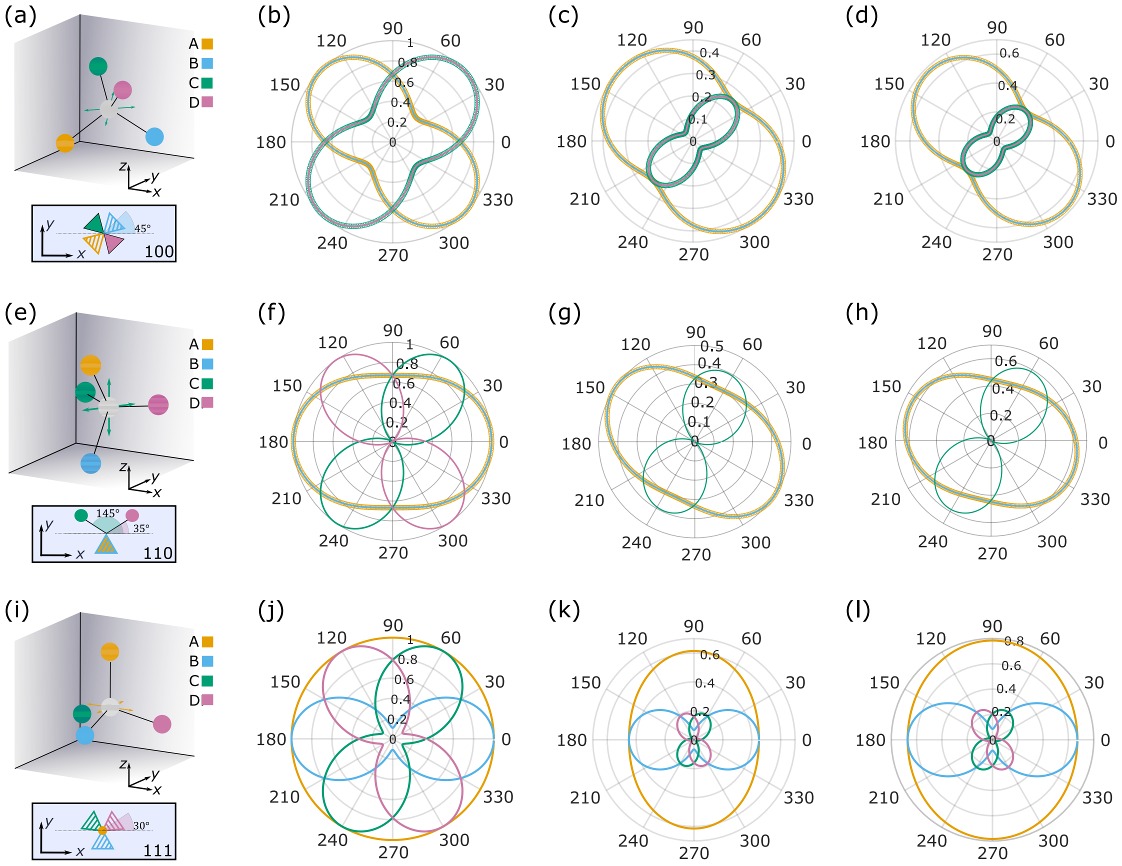

Supplementary figure 1: Simulation results for a laser excitation and PL collection from a {100} (a-d), a {110} (e-h) and a {111} diamond face (i-l).(a) NV centers orientation with respect to a {100} plane (xy plane of the laboratory reference frame). (b) Excitation probability vs laser polarization or, equivalently, PL emission efficiency for different polarization angles. (c) R_i for different PL polarization angles when the laser field is oriented along the projection of family A and B on the {100} plane. (d) χ_i for different PL polarization angles considering the same laser configurations of (c).(e) NV centers orientation with respect to a {110} plane. (f) Excitation probability vs laser polarization or, equivalently, PL emission efficiency for different polarization angles. (g) R_i for different PL polarization angles when the laser field is oriented along family D. (h) χ_i for different PL polarization angles considering the same laser configurations of (g).(i) NV centers orientation with respect to a {111} plane. (j) Excitation probability vs laser polarization or, equivalently, PL emission efficiency for different polarization angles. (k) R_i for different PL polarization angles when the laser field is oriented along the x axis of the laboratory reference frame (l) χ_i for different PL polarization angles considering the same laser configurations of (k).

Concerning the excitation and PL detection from a {111} plane (Supplementary figure 1i), the family perpendicular to that plane (A) has a polarization-independent behaviour [1]. The other three families, due to the NV center C_{3V} symmetry, show a similar plot rotated by 120° (Supplementary figure 1j). Supplementary figure 1k shows the dependence of R_i on the orientation of the polarizer axis when the laser is polarized along the x axis of the reference frame. We observe that the contribution of family A to the total PL increases up to 60% when the polarizer axis is perpendicular to the laser field. Since we showed that the excitation and PL emission of A are independent from both the laser polarization and the polarizer orientation, this simulation demonstrates that the gain in magnetic sensitivity achieved by family A is only due to the suppression of the PL emitted by the three other families. Finally, also in this case, χ_i (Supplementary figure 1l) follows the behaviour of R_i.

2 Contrast of each ODMR peak

We plot here the contrast C_i of each of the eight ODMR peaks obtained by the fitting procedure described in Appendix C. Error bars correspond to errors on the fit parameter related to the contrast with a confidence interval of 95%. As described in fig.2 of the main text, the laser excites NV centers through a diamond {110} plane and the PL is collected from a {100} plane. Supplementary figure 2a shows the ODMR contrast for different angles of the polarizer when the laser is polarized along a $\langle 100 \rangle$ direction. Supplementary figure 2b shows the ODMR contrast for different angles of the half-wave plate when the polarizer axis is set so as to maximize the ODMR contrast of families B and D in Supplementary figure 2a. The ODMR contrast of the two spin transitions ($|0\rangle \rightarrow |\pm 1\rangle$) is the same for family A and B. Larger differences between the contrast of the two spin transitions are observed for family C and D due to the higher transverse component of static magnetic field they are submitted compared to family A and B [2]. The transverse component of the static magnetic field is also responsible of the larger contrast of family D with respect to the other families [3]. Data plotted in fig. 2 are in really good agreement with data and simulations plotted in fig. 3 of the main text, justifying the approximation done in Eq.8 of the main text concerning the magnetic field dependence of the PL emission rates.

References

[1] Naofumi Abe, Yasuyoshi Mitsumori, M. P. Sadgrove, and Keiichi Edamatsu. Dynamically unpolarized single-photon source in diamond with intrinsic randomness. *Scientific Reports*, 7, 2017.

[2] J-P Tetienne, L Rondin, P Spinicelli, M Chipaux, T Debuisschert, J-F Roch, and V Jacques. Magnetic-field-dependent photodynamics of single nv defects in diamond: an application to qualitative all-optical magnetic imaging. *New Journal of Physics*, 14:103033, 10 2012.

[3] E. Moreva, E. Bernardi, P. Traina, A. Sosso, S. Ditalia Tchernij, J. Forneris, F. Picollo, G. Brida, Ž. Pastuović, I. P. Degiovanni, P. Olivero, and M. Genovese. Practical applications of quantum sensing: A simple method to enhance the sensitivity of nitrogen-vacancy-based temperature sensors. *Phys. Rev. Applied*, 13:054057, May 2020.

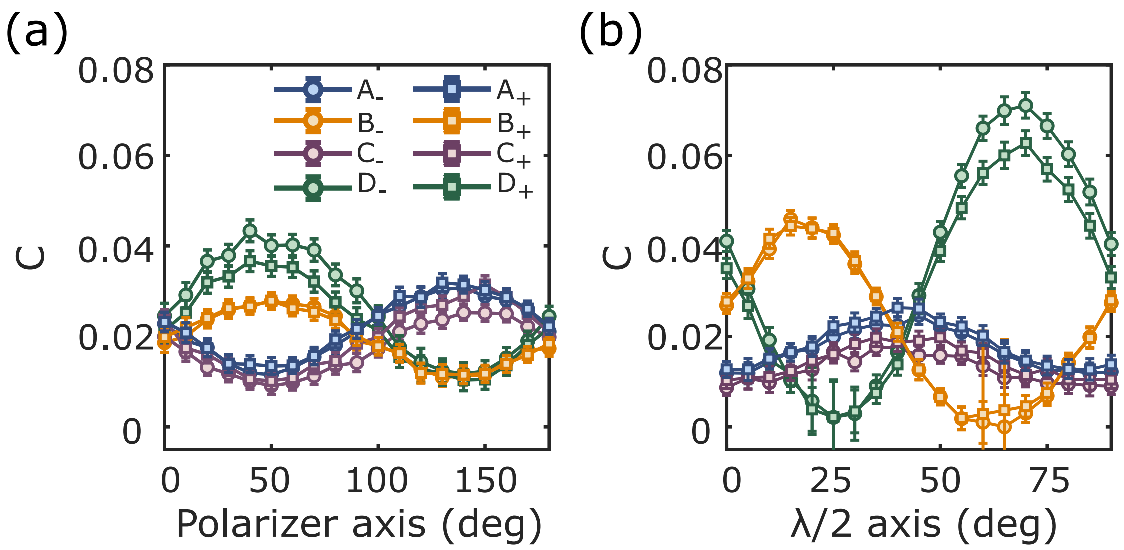

Supplementary figure 2: ODMR contrast of each of the eight ODMR peaks when tuning the polarizer axis (a) and the laser polarization (b). As in the main text, we named the NV center families A, B, C, D being A and D the families whose $|0\rangle \rightarrow |-1\rangle$ transitions resonate respectively at the lowest and the highest frequency. Subscripts + and - identify respectively the $|0\rangle \rightarrow |+1\rangle$ and $|0\rangle \rightarrow |-1\rangle$ transitions.
